# Metastatic Pattern of Truncal and Extremity Leiomyosarcoma: Retrospective Analysis of Predictors, Outcomes, and Detection

**DOI:** 10.3390/jpm12030345

**Published:** 2022-02-24

**Authors:** Seth S. Tigchelaar, Christopher Frey, Dharshan Sivaraj, Nicole A. Segovia, David G. Mohler, Robert J. Steffner, Raffi S. Avedian

**Affiliations:** Department of Orthopedic Surgery, Stanford University, Stanford, CA 94305, USA; chris1fr@stanford.edu (C.F.); ds311@stanford.edu (D.S.); nsegovia@stanford.edu (N.A.S.); dmohler@stanford.edu (D.G.M.); rsteffner@stanford.edu (R.J.S.); ravedian@stanford.edu (R.S.A.)

**Keywords:** leiomyosarcoma, metastasis, extremities

## Abstract

Leiomyosarcomas (LMS) are a heterogenous group of malignant mesenchymal neoplasms with smooth muscle origin and are classified as either non-uterine (NULMS) or uterine (ULMS). Metastatic pattern, prognostic factors, and ideal staging/surveillance studies for truncal and extremity LMS have not been defined. A retrospective analysis of patients diagnosed with histopathology-confirmed truncal or extremity LMS between 2009 and 2019 was conducted. Data collected included demographics, tumor characteristics, staging, surveillance, and survival endpoints. The primary site was defined as: (1) extremity, (2) flank/Pelvis, or (3) chest wall/Spine. We identified 73 patients, 23.3% of which had metastatic LMS at primary diagnosis, while 68.5% developed metastatic disease at any point. The mean metastatic-free survival from primary diagnosis of localized LMS was 3.0 ± 2.8 years. Analysis of prognostic factors revealed that greater age (≥50 years) at initial diagnosis (OR = 3.74, *p* = 0.0003), higher tumor differentiation scores (OR = 12.09, *p* = 0.002), and higher tumor necrosis scores (OR = 3.65, *p* = 0.026) were significantly associated with metastases. Older patients (≥50 years, OR = 4.76, *p* = 0.017), patients with larger tumors (≥5 cm or ≥10 cm, OR = 2.12, *p* = 0.02, OR = 1.92, *p* = 0.029, respectively), higher differentiation scores (OR = 15.92, *p* = 0.013), and higher necrosis scores (OR = 4.68, *p* = 0.044) show worse survival outcomes. Analysis of imaging modality during initial staging and during surveillance showed greater tumor detection frequency when PET imaging was employed, compared to CT imaging (*p* < 0.0001). In conclusion, truncal and peripheral extremity LMS is an aggressive tumor with high metastatic potential and mortality. While there is a significant risk of metastases to lungs, extra-pulmonary tumors are relatively frequent, and broad surveillance may be warranted.

## 1. Introduction

Leiomyosarcomas (LMS) are a heterogenous group of malignant mesenchymal neoplasms with smooth muscle origin typically divided into non-uterine (NULMS) and uterine (ULMS) classifications. LMS is one of the most common subtypes of soft-tissue sarcomas (STS) with an incidence of 1.2 cases per 100,000 person-years and represents between 10% and 20% of all newly diagnosed STS [1]. In general, the overall prognosis for leiomyosarcomas is poor with a reported 5-year survival rate of 35% across all grades [2]. Incidence of LMS increases with age and shows worse prognosis in patients over 50 years old [3,4]. Specifically, LMS can arise in any smooth muscle location, with common sites including the retroperitoneum (20–67% of cases), and peripheral soft tissues (12–41%) including the extremities, skin, and head/neck [2]. To date, there are a small number of studies describing the metastatic rate and pattern for ULMS [4,5,6,7], however the metastatic characteristics for truncal and extremity LMS are not well defined.

Management of LMS includes staging studies to assess metastasis and prognosis at the time of initial diagnosis. Treatment is centered around surgical resection with or without radiation therapy for localized disease and chemotherapy is utilized in select circumstances or in established metastatic disease. Patients with no evidence of disease after initial treatment are followed with surveillance imaging given the high risk of developing metastatic disease (40–80%) [2,4,8,9]. Early detection of metastatic disease has several potential advantages, including initiation of palliative therapy, avoiding progression of occult metastasis to the point of severe pain or other morbidity, patient counseling, and comfort planning. Patients with known metastatic disease are followed with periodic scans to assess response to treatment and tumor stability. While the role of imaging of LMS is pivotal, there is a paucity of evidence-based recommendations on best practices for staging and surveillance imaging of truncal and extremity LMS.

We therefore sought to answer three questions: (1) The anatomical distribution and frequency of metastatic disease in truncal/extremity LMS; (2) Whether factors such as age at primary diagnosis, tumor grade, tumor size, and primary tumor location are associated with metastatic risk or overall survival after primary diagnosis of truncal/extremity LMS; (3) Whether imaging modality is associated with a greater frequency of metastatic disease detection.

## 2. Materials and Methods

In this institutional review board-approved retrospective study, the electronic medical records of patients diagnosed with histopathology-confirmed truncal or extremity LMS at our cancer center between 2009 and 2019 were reviewed. LMS was diagnosed based on results from immunohistochemical markers—including SMA, Desmin, CD34, S100, SOX10, B-catenin, and Pan-cytokeratin—as well as light microscopy and H&E staining. Exclusion criteria included uterine, retroperitoneal, head and neck, cutaneous tumor origin, and patients with incomplete records or surveillance/follow-up.

### 2.1. Patient Demographics, Presentation, and Treatment

A total of 73 patients met inclusion criteria for truncal or extremity LMS, 30 (41.0%) of which were female (Table 1). Four patients were lost to follow-up and at a mean follow-up period of 40.4 months (range, 4.1–126.4 months), 53 patients were alive. The mean age at initial diagnosis was 58.2 ± 14.3 years (range, 17–87 years), with 55 (75.3%) patients ≥ 50 years of age. The mean primary tumor size was 8.4 cm (range, 1.0–23.0 cm) measured as the longest dimension, with 20 (27.8%) tumors < 5 cm in size, 29 (40.3%) tumors between 5 and 10 cm, and 23 (31.9%) tumors ≥ 10 cm in size. The most common primary tumor site was peripheral extremity (50 patients, 68.0%), followed by flank/pelvis (13 patients, 18.0%), and chest wall/spine (10 patients, 14%). Pathology-reported tumor characteristics—including tumor differentiation score, mitotic index, and tumor necrosis score—are reported in Table 1. There were 7 (10.0%) patients with FNCLCC tumor grade I, 24 (34.0%) patients with tumor grade II, and 39 (56.0%) patients with tumor grade III. Resected tumor margins were positive in 21/66 (31.8%) of the resected tumors. Patients were treated with either neoadjuvant therapy, surgical resection, adjuvant chemo- or radiotherapy, or a combination of each. There were 14 (19.2%) patients that received neoadjuvant therapy, and 67 (91.8%) patients that underwent surgical resection.

### 2.2. Variables, Outcome Measures, Data Sources, and Bias

Clinicopathologic data included age at first diagnosis, sex, primary tumor site, tumor size, tumor grade (Federation Nationale des Centres de Lutte Contre le Cancer (FNCLCC) system), presence of metastatic disease at diagnosis, number of metastatic sites at first presentation, initial staging imaging modality, treatment type, presence of local recurrence, time to metastasis, survival after primary diagnosis, and surveillance/treatment response imaging modalities. Primary site was defined as: (1) peripheral extremity, (2) flank/pelvis, or (3) chest wall/spine. Recurrent or metastatic disease was confirmed either by biopsy or by the presence of progression on serial surveillance imaging.

### 2.3. Statistical Analysis

Patient demographics are presented as means and range for continuous variables and by frequency and percentage for categorical variables. Metastatic-free survival and overall survival were calculated as the interval from the date of primary diagnosis to detection of metastatic disease, or date of death, respectively. The prognostication of age, tumor grade (evaluated by its individual components: tumor differentiation score, mitotic index, and tumor necrosis score), margin status, and tumor size on metastatic prognostication and survival was evaluated in patients presenting without metastatic disease. The Kaplan–Meier method, log-rank test, multivariable Cox proportional hazards regression models, and Chi-Square tests were used for estimation, testing, and multivariable modeling of overall survival. Analyses were considered significant with a *p*-value < 0.05. All analyses were done using RStudio version 1.1.456 (Boston, MA, USA).

## 3. Results

### 3.1. Anatomical Distribution and Frequency of Metastatic Disease in Truncal/Extremity LMS

**Figure 1:**
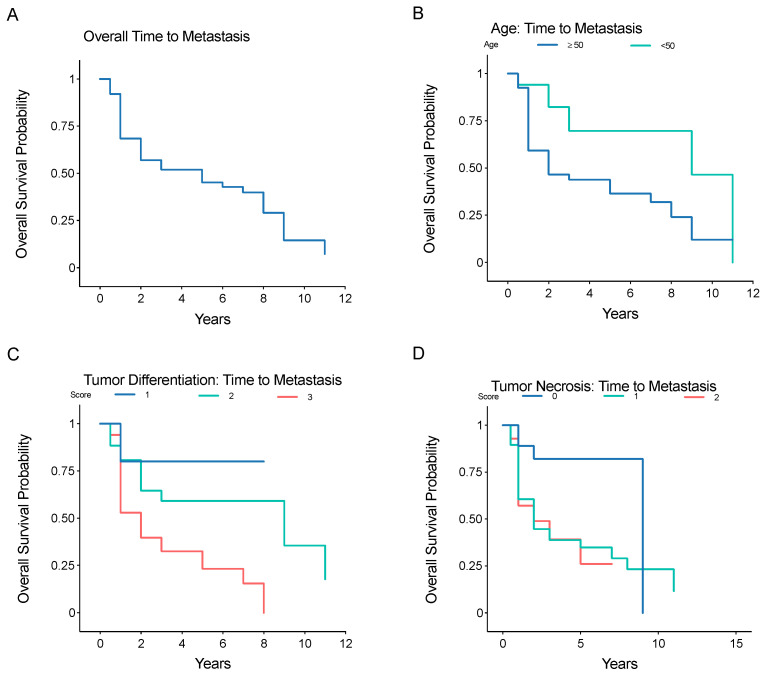
Kaplan–Meier analysis of metastatic-free survival after initial diagnosis of localized LMS, (**A**) overall time to metastasis, or stratified by (**B**) age greater or less than 50 years, (**C**) pathologist-reported tumor differentiation score (1, 2, or 3), or (**D**) pathologist-reported tumor necrosis score (0, 1, or 2).

At the time of primary diagnosis, 17 (23.3%) patients had metastatic disease. Of the 56 patients that did not have metastatic disease at the time of primary diagnosis, 33 (58.9%) subsequently developed metastases. A total of 50 (68.5%) patients had metastatic disease develop at any point in the mean follow-up period of 40.4 months (range, 4.1–126.4 months) (Table 1). In patients with primary tumors classified as grade I, II, or III, there was metastatic progression in 1 (14.3%), 17 (70.8%), and 32 (82.1%) patients, respectively. The mean tumor size was 8.35 cm (Range, 1–23). The mean metastatic-free survival from primary diagnosis of localized LMS of any grade was 3.0 ± 2.8 years (range, 0.5–11). The mean metastatic-free survival from primary diagnosis for patients with grade III LMS was 1.6 ± 1.7 years (range, 0.5–8). The rate of metastatic disease after primary diagnosis in patients without initial metastases, and not including local recurrence, was 32% at 1 year, 55% at 5 years, and 93% at 11 years (Figure 1A). The rate of metastatic disease was significantly higher in older patients (age ≥ 50 years) (Figure 1B, *p* = 0.024), patients with higher tumor differentiation scores (Figure 1C, *p* = 0.0019) (based on pathology report), and patients with higher tumor necrosis scores (Figure 1D, *p* = 0.032).

Amongst the 50 patients with metastatic disease, the most common site of metastasis was lung (42 patients, 84.0%), followed by abdomen/thorax/visceral organs (26 patients, 52.0%), bone (17 patients, 34.0%), skin/soft tissue (14 patients, 28.0%), lymph nodes (3 patients, 6.0%), brain (2 patients, 4.0%), and vessels (1 patient, 2.0%) with 30 (41.1%) patients having metastatic disease in three or more of these sites (Table 1). While the lungs were one of the most common anatomical sites for metastases, there were a significant number of metastatic sites outside of the lungs—in 22 (44.0%) patients, the first site of detected metastatic disease was not in the lungs, highlighting the need for broad surveillance beyond thoracic-focused imaging. Following surgical resection of primary tumors, local recurrence occurred in 24/66 (36.4%) patients. Surgical margins, as documented in the pathology report, were positive in 21/66 (31.8%) of patients, and negative in 45 (68.3%) of patients. In those patients with recurrence, 12 (50%) had pathology reports documenting positive margins. Margin status (positive or negative) was not significantly associated with local recurrence.

### 3.2. Prognostic Factors Associated with Metastatic Risk or Overall Survival after Primary Diagnosis of Truncal/Extremity LMS

Univariate and multivariate analyses of metastatic prognosticators revealed that age at initial diagnosis (≥50 years), tumor differentiation score and tumor necrosis score (but not mitotic index), and the use of adjuvant therapy were significantly associated with the development of metastatic disease (Table 2). Patients aged 50 and above had a significantly higher rate of metastatic disease compared to patients under the age of 50 (Multivariate analysis: odds ratio (OR) = 2.54, *p* = 0.001), with 42/55 patients ≥ 50 years old developing metastatic disease, compared to 8/18 patients < 50 years old. Tumor differentiation and necrosis scores were significantly associated with the development of metastatic disease. Additionally, tumor differentiation scores of 2 and 3 showed a significant predilection for metastasis to the lungs (Multivariate analysis: OR = 5.22, *p* = 0.039, OR = 11.76, *p* = 0.004, respectively). Tumor sizes of 5–10 cm, ≥10 cm (multivariate analysis: *p* = 0.551, *p* = 0.319, respectively), primary tumor location (*p* = 0.355), or tumor margin status were not associated with development of metastatic disease.

**Figure 2:**
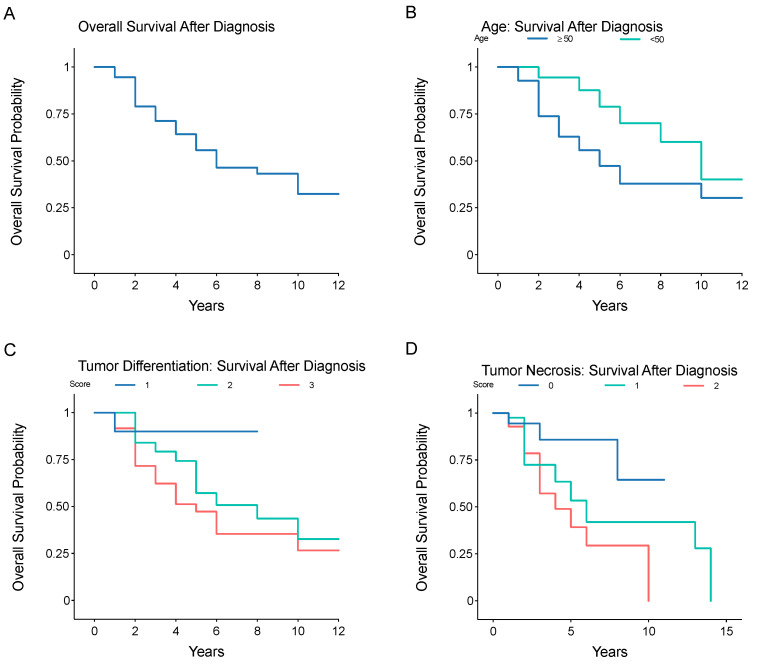
Kaplan–Meier analysis of overall survival, stratified by tumor grade I, II, or III, after initial diagnosis of localized LMS. (**A**) overall survival, and survival stratified by (**B**) age greater or less than 50 years, (**C**) pathologist-reported tumor differentiation score (1, 2, or 3), or (**D**) pathologist-reported tumor necrosis score (0, 1, or 2).

**Figure 3:**
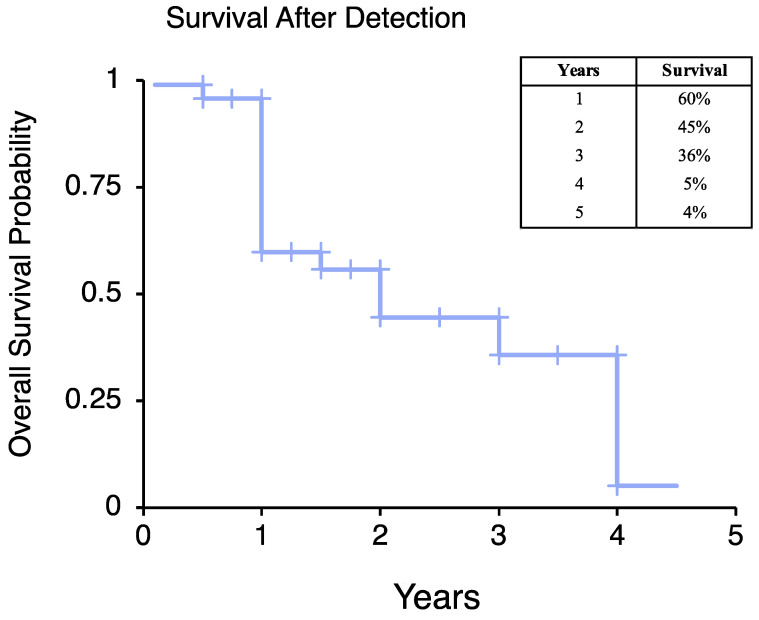
Kaplan–Meier analysis of overall survival after detection of metastatic disease.

The mean overall survival after primary diagnosis was 4.7 ± 3.3 years (range, 1–14 years) (Table 1), with a survival rate after primary diagnosis of 55% at 5 years, 43% at 9 years, and 32% at 13 years (Figure 2A). Survival was significantly reduced in older patients ((≥50 years) (Figure 2B, *p* = 0.029), patients with higher tumor differentiation scores (Figure 2C, *p* = 0.024), and those with higher tumor necrosis scores (Figure 2D, *p* = 0.043). After detection of metastatic disease, the survival rate was 60% at 1 year, 45% at 2 years, 36% at 3 years, 5% at 4 years (Figure 3). Tumor differentiation, mitotic index, and tumor necrosis scores, components of tumor grading, were assessed for survival prognosis (Table 3). Tumor differentiation scores of 3 (HR = 15.92, *p* = 0.013), and tumor necrosis scores of 1 (HR = 3.52, *p* = 0.041) and 2 (HR = 4.68, *p* = 0.044) showed significantly worse survival. Additionally, older patients (≥50 years) showed worse survival, (HR = 4.76, *p* = 0.017), patients with larger tumors (≥10 cm), (HR = 1.92, *p* = 0.029) and patients with higher tumor necrosis scores of 1 (HR = 3.52, *p* = 0.041) or 2 (HR = 4.68, *p* = 0.044), or tumor differentiation scores of 3 (HR = 15.92, *p* = 0.013). Primary tumor site was not associated with overall survival. Primary tumor location, either flank/pelvis or chest wall/spine, does not influence overall survival (HR = 1.37, *p* = 0.412, HR = 1.73, *p* = 0.174, respectively).

### 3.3. Impact of Imaging Modality on Frequency of Metastatic Disease Detection

A total of 84 imaging studies were performed across 73 patients for initial staging. These included CT scans (*n* = 47, 56.0%), PET/CT (*n* = 25, 30.0%), nuclear bone scan (*n* = 7, 8.3%), and chest X-ray alone (*n* = 5, 6.0%) (Table 4). Comparing the rate of primary tumor detection and metastatic disease at baseline based on the imaging modality used at initial staging, PET/CT imaging was found to be associated with a significantly greater rate of tumor detection, identifying 20 instances of metastatic disease when compared to CT CAP imaging, which identified 14 instances (Chi-square = 16.5, *p* < 0.0001) (Table 5).

During the follow-up period, there were a total of 665 follow-up visits for surveillance/treatment response imaging (Table 4). Imaging studies included CT CAP (*n* = 343, 68%), PET/CT (*n* = 166, 19%), and chest X-rays (*n* = 134, 15.4%), and nuclear bone scan (*n* = 22, 3.2%). On average, patients underwent surveillance imaging every 2.4 months, for an average of 5 visits per year (range, 1–13 visits/year), with an average lifetime total of 11 studies performed (range, 1–51 studies).

We compared the rate of detection of tumor metastases during this period, based on surveillance imaging modality utilized (CT CAP vs. PET/CT). PET/CT imaging detected new tumor metastases significantly more often, when compared to CT CAP (Chi-square = 11.32, *p* < 0.001) (Table 5). An analysis of site-specific detection reveals that PET/CT was associated with significantly more frequent metastatic tumor detection in the abdomen/visceral organs (Chi-square = 8.18, *p* = 0.004), and skin/soft tissue (Chi-square = 9.97, *p* = 0.0016) than CT CAP.

## 4. Discussion

Despite the relatively poor prognosis of LMS, one of the more common soft-tissue sarcoma subtypes, there is a paucity of data describing the metastatic pattern, prognostic factors, and efficacy of specific staging or surveillance imaging modalities [10,11,12,13]. We therefore sought to answer three questions: (1) What is the anatomical distribution and frequency of metastatic disease in truncal/extremity LMS? (2) Are factors such as age at primary diagnosis, tumor differentiation score, necrosis score, or mitotic index (the components of tumor grade), tumor size, and primary tumor location associated with metastatic risk or overall survival after primary diagnosis of truncal/extremity LMS? (3) Is imaging modality associated with a greater frequency of metastatic disease detection? Here, we show that truncal/extremity LMS has a high rate of metastasis, with significantly higher rates in older patients and those with higher tumor differentiation scores and necrosis scores at initial diagnosis. We show that truncal/extremity LMS has a high rate of metastases to extra-pulmonary sites, and the use of PET/CT imaging, both at initial staging and throughout surveillance, was associated with a greater rate of metastatic tumor detection.

This study has several limitations, including its retrospective nature and non-randomized design. As a retrospective study, we were reliant on the accuracy of patient records. Furthermore, as we collected data from our single cancer institution, bias in patient referral should be considered. As a retrospective, non-randomized investigation, this study was not designed to parse out the utility of each imaging modality for surveillance, or progression of disease/response to treatment. As such, this study was unable to assess the duration or specific therapeutic regimes used, due to limited information in patient charts. The use of imaging modalities is often influenced by factors such as patient insurance or provider decision.

In this retrospective analysis, we identified 73 patients with truncal or extremity LMS. Consistent with the findings of others, the lungs were the most common metastatic site [6,7], with a relatively high rate of metastatic disease arising in the abdomen, thorax, visceral organs, bone, and skin or soft tissues. We show that tumors with differentiation scores of 2 and 3 are significantly more likely to metastasize to the lungs, with an odds ratio of 8.32 (*p* = 0.026) and 14.65 (*p* = 0.003) for scores of 2 or 3, respectively. The 5-year overall survival rate in this cohort was 59%, while Shoushtari et al. reported a 5-year survival rate of under 25% in an analysis of only metastatic LMS, and Lamm et al. reported a 5-year survival rate of 44.4% [6,7]. The lower 5-year survival rates previously reported likely represent the difference in proportions of high vs. low-grade LMS. The largest study focusing on LMS to date, conducted by Shoushtari and colleagues in 2016, provides a description of the overall survival and response to systemic therapy in extrauterine metastatic LMS [6] but lacks a precise description of metastatic pattern. Furthermore, in 2014, Lamm et al. compared uterine to non-uterine LMS, showing that lungs are the most common metastatic site in both uterine and non-uterine LMS, with initial metastatic disease serving as a prognostic factor for overall survival [7]. More recently, Lee et al. compared the response to radiation treatment of truncal/extremity LMS versus non-LMS soft-tissue sarcomas. Lee and colleagues describe comparable average age and tumor size of 63 years and 6.0 cm, respectively [11]. Similarly, Gladdy et al. describe a median age of 57 years, an average tumor size of 6.0 cm, and identified high grade tumors as predictive of disease-specific survival [10].

In this study, 10.0% of patients were classified as grade I, compared to 3% and 2% in the studies by Shoushtari et al. and Lamm et al. [6,7]. In our analysis of metastatic prognosticators, we identified tumors with high differentiation scores (2, 3), high tumor necrosis scores, and patients over the age of 50 showed significantly greater rates of metastasis, consistent with current reports of ULMS and NULMS [4,5,6,7]. We show that tumor size is not associated with metastatic risk (OR = 1.04, *p* = 0.515), but that tumors sized 5–10 cm (HR = 2.12, *p* = 0.020), as well as tumors >10 cm (HR = 1.92, *p* = 0.029) are associated with survival. Interestingly, previous reports by Shoushtari et al. and Tirumani et al. both found no association between size and time to metastatic disease or survival, despite its role in FIGO staging [4,6]. The lack of prognostication of size on metastatic disease may be related to the heterogeneity in primary tumor locations included in this study—since we were focused primarily on LMS in the extremities, the variability in size may make metastatic prognostication challenging. In future work, we seek to perform subgroup analyses on primary tissue sites to see if size plays a larger role in outcomes. While size did have prognostic value in survival time, this may reflect the larger sizes of the metastatic tumors themselves, relating to worse outcomes. Furthermore, primary tumor location—either flank/pelvis or chest wall/spine—does not influence overall survival (HR = 1.37, *p* = 0.412, HR = 1.73, *p* = 0.174, respectively).

Effective detection, diagnosis, and surveillance are vital in the treatment and management of LMS. Analyses of efficacy of radiological staging and surveillance modalities for LMS are also sorely needed. Here, we found a significantly worse overall survival in patients that underwent more frequent radiological surveillance. We believe this represents positive clinical decision-making, such that patients that were deemed to have more aggressive tumors at diagnosis subsequently underwent more frequent surveillance. While Chest CT with or without contrast remains the benchmark for assessing lung metastases, there is conflicting evidence for obtaining an abdomen/pelvis CT for staging [9]. Reports of incidence rates of metastatic disease in the abdomen or pelvis vary—one report from a single institution suggests a 16.0% incidence rate [14], supporting routine abdomen/pelvis CT, while another retrospective review reported only a 2.9% incidence rate; this would argue against routine use of abdomen/pelvis CT for staging and monitoring in the setting of soft-tissue sarcoma of the extremity [15]. There is also a growing role for PET/CT for staging, surveillance, and gauging treatment response of soft-tissue sarcomas [16,17,18]. In two studies of LMS, tumor ^18^F-FDG uptake—as measured by the maximum standardized uptake value (SUV_max_)—was a powerful prognostic factor for overall survival correlating with tumor grade and size [17,19]. In this study, we show that the risk of metastatic disease to any extra-pulmonary area after primary LMS diagnosis occurred in 36/73 patients (49.3%). Of those 50 patients that developed metastatic disease, 35 (72.0%) developed metastatic sites outside of the lungs, supporting the use of imaging that extends beyond routine chest CT. Despite the potential strength of PET/CT imaging, there have been relatively few reported series, with low case numbers to justify the routine application of PET/CT imaging of LMS. In this study, we provide the largest comparison known to date of metastatic tumor detection between PET/CT imaging and CT CAP in a cohort of patients with truncal/extremity LMS.

In comparing the rate of tumor detection between the two most common and comparable imaging modalities, CT CAP and PET/CT, we found that PET/CT imaging detected significantly more tumors both at initial staging (Chi-square = 4.7, *p* = 0.03), and throughout surveillance/treatment response (Chi-square = 11.32, *p* < 0.001). In this study, we show that metastases to extra-pulmonary sites are relatively frequent, suggesting a particularly important utility for PET/CT imaging in detecting metastatic disease at sites that are not readily detected on conventional CT imaging. In particular, PET/CT imaging had a greater rate of detecting tumors in the abdomen/visceral organs, and skin/soft tissue—areas that might be missed using CT chest/abdomen/pelvis imaging. However, caution is warranted as Hensley et al. have suggested that PET has not been shown to be superior for staging of uterine and ovarian LMS, and may miss small volume lung metastatic tumors, often necessitating chest CT imaging in conjunction with whole body PET/CT imaging [20]. Moreover, our study was not designed to parse out the utility of each imaging modality for surveillance or progression of disease/response to treatment. The use of PET/CT versus CT CAP was not always dictated by care algorithms. Further investigations are needed to elucidate the clinical benefit of specific staging and surveillance techniques and timing.

## 5. Conclusions

Truncal and peripheral extremity LMS is an aggressive tumor with high metastatic potential. Notably, over 30% of all primary metastatic sites were not in the lungs, suggesting thorough staging and surveillance imaging beyond the lungs is warranted. Tumors with differentiation scores of 2 or 3, as well as tumors with high necrosis scores, carry a high-risk of developing metastatic disease. Five- and 10-year overall survival rates are low, particularly in older patients, with larger tumors. Historically, CT scans have been primarily used for initial staging and disease surveillance/treatment response, however in our study PET/CT scans were found to detect overall metastatic sites more frequently and were not inferior to CT CAP in detecting lung metastases in our series. This suggests that PET/CT may be preferred over conventional CT for staging and surveillance of LMS. Further investigations into the optimal imaging techniques for LMS staging and surveillance are needed.

## Figures and Tables

**Table 1:** Patient Demographics and Tumor Characteristics.

Variable	Factor	*n*, Mean	%, Range
Gender	Female	30	41%
	Male	43	59%
Mean Age (Years)	≥50 years	55	75%
	<50 years	18	25%
Tumor Size (cm)	<5 cm	20	28%
	5–10 cm	29	74%
	≥10 cm	23	32%
Primary Tumor Site	Extremity	50	68%
	Flank/Pelvis	13	18%
	Chest Wall/Spine	10	14%
Tumor Differentiation Score	1	10	14%
	2	26	36%
	3	36	50%
Mitotic Index	1	26	36%
	2	29	40%
	3	17	24%
Tumor Necrosis Score	0	18	25%
	1	40	56%
	2	14	19%
Total Score	2	5	7%
	3	2	3%
	4	18	25%
	5	6	8%
	6	32	44%
	7	9	13%
	8	0	0%
Histological Grade	I	7	10%
	II	24	34%
	III	39	56%
Pathologist-Reported Margins	Positive	21	32%
	Negative	45	68%
Presentation Status	Primary Disease	56	77%
	Metastatic Disease	17	23%
Treatment	Neoadjuvant Therapy	14	19%
	Surgical Resection	67	92%
	Adjuvant Chemotherapy	25	34%
	Adjuvant Radiotherapy	28	38%
	Adjuvant Chemotherapy + Radiotherapy	15	21%
Development of Metastatic Disease by Histological Grade	I	1	14%
	II	17	71%
	III	32	82%
Time to Metastatic Disease from Diagnosis (Years)	Any Location	50	69%
	Lung	42	84%
	Abdomen/Thorax/Visceral Organ	26	52%
	Bone	17	34%
	Skin/Soft Tissue	14	28%
	Lymph Node	3	6%
	Brain	2	4%
	Vessel	1	2%
	Yes	24	36%
	No	42	64%
Mean Survival After Primary Diagnosis, All Grades (Years)		4.7	1–14

**Table 2:** Univariate and Multivariate Analysis of Prognostic Factors for Metastatic Disease. * indicate significant results, *p*-value < 0.05.

		Univariate		Multivariate	
Variable	Level	OR (95% CI)	*p*-Value	OR (95% CI)	*p*-Value
Any Metastasis	Age ≥ 50 years	3.74 (1.32–12.36)	<0.001 *	2.54 (1.18–8.98)	0.001 *
	Positive Margins	1.12 (0.42–2.29)	0.892	1.02 (0.52–1.49)	0.921
	Tumor Differentiation				
	2	4.01 (0.71–22.61)	0.116	3.23 (0.82–12.36)	0.223
	3	14.02 (2.54–79.65)	0.003 *	12.09 (2.29–67.42)	0.002 *
	Mitotic Index				
	2	1.39 (0.47–4.15)	0.552	1.17 (0.78–2.51)	0.673
	3	0.83 (0.24–2.82)	0.759	0.92 (0.22–2.17)	0.889
	Tumor Necrosis				
	1	6.85 (1.98–23.76)	0.002 *	4.12 (1.42–13.34)	0.032 *
	2	4.68 (1.04–21.04)	0.044 *	3.65 (0.80–16.51)	0.026 *
	Tumor Size				
	5–10 cm	1.02 (0.94–1.17)	0.515	1.01 (0.96–1.12)	0.551
	≥10 cm	1.73 (0.83–2.09)	0.221	1.34 (0.81–1.78)	0.319
Lung Metastasis	Tumor Differentiation				
	2	8.32 (1.87–187.36)	0.026 *	5.22 (1.65–89.32)	0.039 *
	3	14.65 (1.29–237.42)	0.003 *	11.76 (1.04–143.29)	0.004 *

**Table 3:** Multivariate Analysis of Prognostic Factors for Overall Survival. * indicate significant results, *p*-value < 0.05.

Variable	Level	Hazard Ratio (95% CI)	*p*-Value
Age	≥50 years	4.76 (0.06–0.75)	0.017 *
Primary Site	Extremity	0.59 (0.29–1.19)	0.140
	Flank/Pelvis	1.37 (0.65–2.90)	0.412
	Chest Wall/Spine	1.73 (0.78–3.82)	0.174
Tumor Margins	Positive	1.32 (0.61–2.01)	0.424
Tumor Differentiation	2	9.02 (0.99–81.58)	0.051
	3	15.92 (1.81–140.17)	0.013 *
Mitotic Index	2	1.91 (0.65–5.60)	0.239
	3	0.82(0.24–2.81)	0.748
Tumor Necrosis	1	3.52 (1.05–11.76)	0.041 *
	2	4.68 (1.04–21.04)	0.044 *
Tumor Size	5–10 cm	2.12 (0.66–6.78)	0.020
	≥10 cm	1.92 (1.31–4.22)	0.029 *
Surveillance Frequency	(≤4 mo)	2.72 (1.17–4.79)	0.010 *

**Table 4:** Radiological Staging and Surveillance Frequency.

Variable	*n*	%
**Initial Staging Studies**		
CT Chest/Abdomen/Pelvis	47	56%
PET/CT	25	30%
Nuclear Bone Scan	7	8%
Chest X-ray	5	6%
**Radiological Surveillance Studies**		
CT chest/abdomen/pelvis	343	52%
PET/CT	166	25%
Chest X-ray	134	20%
Nuclear Bone Scan	22	3%
**Mean Annual Surveillance Frequency (Months)**	5	1–13
**Mean Number of Lifetime Surveillance Scans**	11	1–51

**Table 5:** Univariate Analysis of Frequency of Tumor Detection by Radiological Modality. * indicate significant results, *p*-value < 0.05.

Imaging Paradigm	Imaging Modality	Frequency Used	Frequency of Tumor Detection	Chi-Square Statistic	*p*-Value
Initial Staging	CT CAP	47	14	16.5	<0.0001 *
	PET/CT	25	20		
Radiological Surveillance	CT CAP	343	24	23.2	<0.0001 *
	PET/CT	166	36		
Staging + Surveillance	CT CAP	390	38	36.2	<0.0001 *
	PET/CT	191	56		

## Data Availability

The data that support the findings of this study are available from the corresponding author upon reasonable request.
